# Gemcitabine-incorporated polyurethane films for controlled release of an anticancer drug

**DOI:** 10.1186/s40824-019-0169-7

**Published:** 2019-11-20

**Authors:** Seong Hoon Choi, Il-Hoon Cho, Sangsoo Park

**Affiliations:** 1 Inbody Research Center, Gangnam-gu, Seoul, 06313 South Korea; 2 Department of Biomedical Laboratory Science, College of Health Science, Eulji University, Seongnam, Gyeonggi-do 13135 South Korea; 3 Department of Biomedical Engineering, College of Health Science, Eulji University, 553 Sanseongdae-ro, Sujeong-gu, Seongnam-si, Gyeonggi-do 13135 Republic of Korea

**Keywords:** Gemcitabine, Anticancer drug, Drug-releasing stent, Polyurethane coating, Stent coating

## Abstract

**Background:**

Local delivery of anti-cancer drugs through a stent is a very promising and anticipated treatment modality for patients who have obstructions in their gastrointestinal tract with malignant tumors. Anticancer drug release via stents, however, needs to be optimized with respect to drug delivery behavior for the stents to be effective for prolonged containment of tumor proliferation and stent re-obstruction. Local stent-based drug delivery has been tested using an effective anti-cancer drug, gemcitabine, but the release from the stent-coated polyurethane films is often too fast and the drug is depleted from the coated film virtually in a day.

**Methods:**

To moderate the drug release from a polyurethane film, a gemcitabine-incorporated polyurethane film was enveloped with a pure polyurethane film, with no drug loading, and with a silicone film by solution casting after activation of the silicone film surface with plasma treatment.

**Results:**

The pure polyurethane barrier film was effective; the interface of the two were indistinguishable on scanning electron microscopy, and the initial burst, i.e., the cumulative release in a day, decreased from 90 to 26%. The silicone film barrier, on the other hand, was defective as voids were seen using a scanning electron microscope, and micro-separation of the two layers was observed after the film was immersed in phosphate-buffered saline for 1 day during the in vitro drug release study.

**Conclusions:**

Enveloping a gemcitabine-releasing polyurethane film with a homo-polymer barrier film was quite effective for moderating the initial burst of gemcitabine, thus, prolonging the release time of the drug. Enveloping the polyurethane film with a silicone film was also possible after plasma treatment of the silicone film surface, but the two films eventually separated in the aqueous environment. More studies are needed to tune the drug release behavior of gemcitabine from the stent covering film before attempting a clinical application of an anti-cancer drug releasing stent.

## Introduction

Local delivery of antineoplastic agents via a stent coating is considered to be highly desirable, as most of the patients using gastrointestinal stents are in the terminal stage and surgical operation is not recommended [1–6]. Particularly, malignant tumors in the pancreatic duct or biliary tract are known to have an extremely poor prognosis because they are usually detected in advanced stages. Because curative surgery is difficult for most of these patients, there has been an increasing interest in developing effective anti-cancer drug-loaded stents that can be placed in the biliary duct [7–11].

Two anti-proliferative drugs have been studied extensively for potential application in drug-delivery via stents to control the tumor growth around the stent. The most studied drug is paclitaxel, a taxane derived from the bark of a yew tree, *Taxus brevifolia*, which has been shown to exhibit significant activity against a variety of solid tumors when administered systemically. However, the effectiveness of paclitaxel delivery via a stent for tumor suppression could be hampered by its extreme hydrophobicity and limited solubility in water [11]. Gemcitabine, in contrast, is administered in the form of a quaternary ammonium salt that is highly soluble in water, and it is released from the solvent-cast polyurethane (PU) film virtually within a day [12]. A rapid release of gemcitabine from the stent could, however, be a problem because a very high dosage in a short time period could lead to local side-effects on the surrounding tissues. Rapid drug release from the stent could also compromise the effectiveness of the anticancer drug-eluting stent as, if the stent empties its drug load too soon, it will not be able to help control the tumor cell proliferation in the later days. Thus, extensive in vitro and in vivo studies are warranted to optimize the delivery method of gemcitabine from the coated stent before attempting to use the anticancer drug-eluting stent in a clinical setting.

In a previous study, we reported that gemcitabine was released virtually within a day when a clinically available form of the drug was loaded in a polyurethane film and that the release could be prolonged for up to a week when the pure chemical form of gemcitabine was used instead [12]. To moderate the rapid release of gemcitabine from polyurethane film, we tried another approach by placing a barrier film on the top and bottom of the drug-loaded polyurethane film. Herein, we report the preparation of gemcitabine-incorporated polyurethane (GPU) films, enveloped by either pure polyurethane film or room–temperature-cured silicone film, the morphology of these films, and the effects of these barrier films on the drug release behavior of gemcitabine.

## Methods

### Chemicals and reagents

Gemcitabine, with a trade name of Gemzar®, was purchased from Yuhan Co. Ltd. (Seoul, South Korea), and used as it was supplied. Medical grade polyurethane, ChronoFlex AL 85A, was purchased from AdvanSource Biomaterials Corporation (Wilmington, MA, United States) and medical grade silicone NuSil™ MED-6600 was purchased from NuSil Technology LLC (Carpinteria, CA, United States). N, N-dimethylacetamide (DMAc) (KANTO Chem. Co. Ltd., Japan) and tetrahydrofuran (THF) (Junsei Chem. Co. Ltd., Japan) were reagent grade. Cover glass for film preparation was obtained from Deckglaser (Knittel Gläser, Germany).

### Gemcitabine incorporated film preparation

**Fig. 1:**
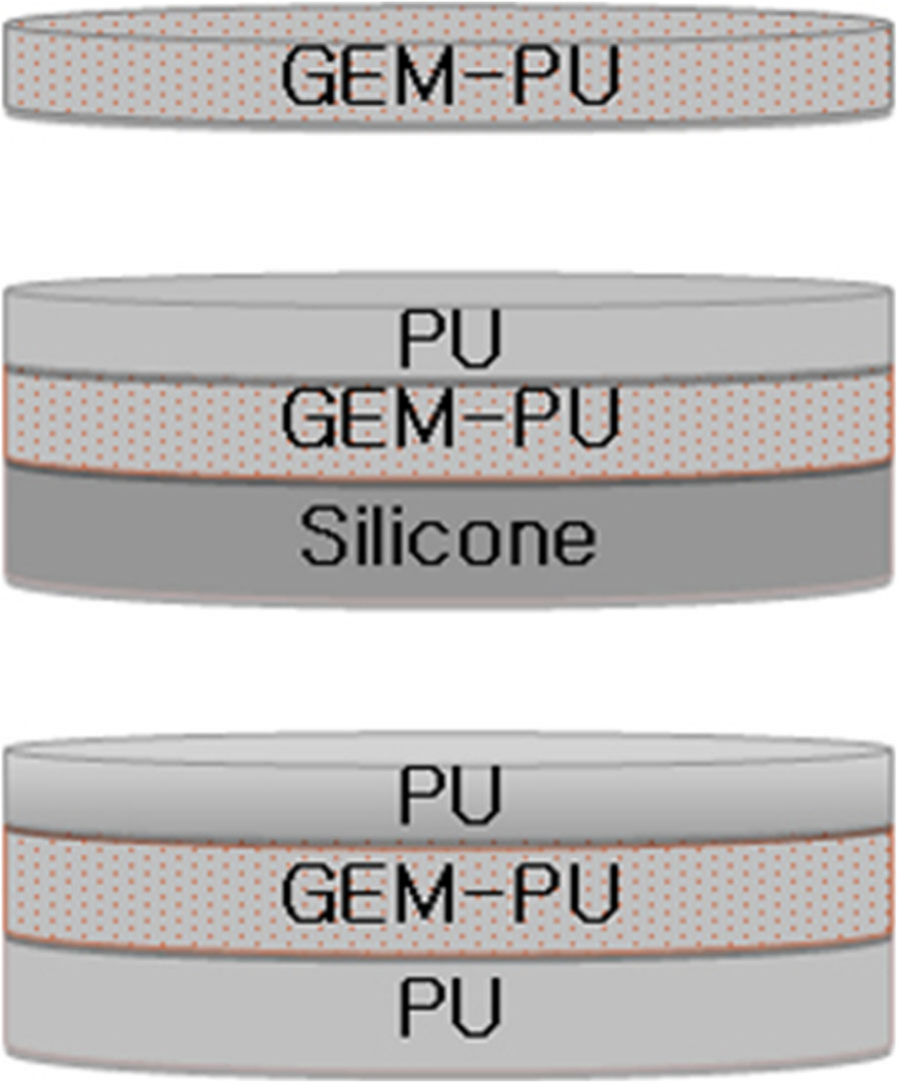
Schematics of the GPU, Silicone-(GPU)-PU, and PU-(GPU)-PU films. GEM-PU represents a gemcitabine-incorporated polyurethane film, PU a polyurethane film without the drug, and Silicone represents a silicone film without the drug

The films were fabricated in a teflon well plate, as describe previously [11]. The wells on the plate had a diameter of 1.5 cm, and the film was prepared in one or three layers, as shown in Fig. 1. The casting solution for the GPU film was prepared by dissolving an appropriate amount of gemcitabine in DMAc and mixing this solution with a premixed 12% (w/v) polyurethane (PU) in DMAc solution. After complete mixing of the two solutions, a gemcitabine-dissolved polyurethane solution was delivered to the teflon wells and the solvent was evaporated thoroughly inside a heating oven at 120 °C. For preparation of the PU-GPU-PU film, which is a gemcitabine-incorporated film sandwiched in between the two pure polyurethane films, the PU film was prepared first and a gemcitabine-dissolved PU solution was delivered onto the wells and cured inside the oven, followed by the formation of another PU film on the top. The bottom silicone layer of the Si-GPU-PU film was prepared by mixing the two solutions of NuSil™ MED-6600. After curing the silicone layer inside a heating oven at 120 °C, the silicone layer was treated with a plasma generator (Plami; APP Inc., Gyeongi-Do, South Korea) to produce adhesive functional groups on the surface of silicone, followed by successive GPU and PU film formations on the top of the silicone film.

### Study of the film morphology

The morphology of the films before and after the drug release study was examined with a field emission electron microscope (Hitachi S-4700, Japan) and a video microscope system ICS-305B (Sometech Vision, Seoul, Korea).

### In vitro drug release study

Gemcitabine loading in GPU films for in vitro release study was 10% of the GPU film (wt/wt). Four samples of GPU, PU-GPU-PU, Si-GPU-PU films were placed inside 50-mL conical tubes, and 10 mL of 0.01 M phosphate-buffered (PBS) was added to each tube. The tubes containing the gemcitabine-incorporated PU films were then placed in a shaking water-bath at 37 °C at 120 rpm. The concentration of gemcitabine in the buffer was determined by HPLC at 1, 3, and 8 h, and at 1, 3, 7, 11, 21 days, and the PBS in each tube was replaced with fresh buffer.

For HPLC determination of gemcitabine concentration, a HP1100 series system (Agilent Technologies, Palo Alto, USA) equipped with G1322A online degasser, G1312A binary pump, G1313A autosampler, G1316A thermostated column compartment, and G1315A diode-array detector was used. Data were acquired and processed with HP Chemstation chromatography manager software. Chromatographic separations were achieved using a C18 column at 25 °C. The mobile phase was sodium acetate buffer (pH 5) used at a flow rate of 1.5 mL/min, and UV detection was performed at 282 nm (Additional file 1: Figure S1).

## Results

The characteristics of the GPU films are presented in Table 1. The average thickness and mass of the GPU films were 21 (±1) μm and 330 (±6) mg, respectively. The drug loadings were calculated from the concentration of gemcitabine in the gemcitabine-polyurethane solution and the volume of the solution added to the teflon well. From the volume ratio of the top PU:GPU:bottom PU solutions (1:2:1), a thickness of 7, 14, and 7 μm was expected for the top PU:GPU:bottom PU films. Because of the high viscosity of silicone solutions, it was difficult to make a thin silicone film and the average thickness of the silicone layer was 25 μm. Thus, the Si-GPU-PU film is expected to be 25, 14, and 7 μm thick in the silicone, GPU, and PU layers, respectively.

**Table 1:** Characteristic of the gemcitabine incorporated films

Film Type	drug loading, μg	Average thickness, μm
GPU	330(±17)	21 (±1)
PU-(GPU)-Si	220(±12)	46 (±3)
PU-(GPU)-PU	220(±12)	28(±2)

### Morphology of the GPU film

**Fig. 2:**
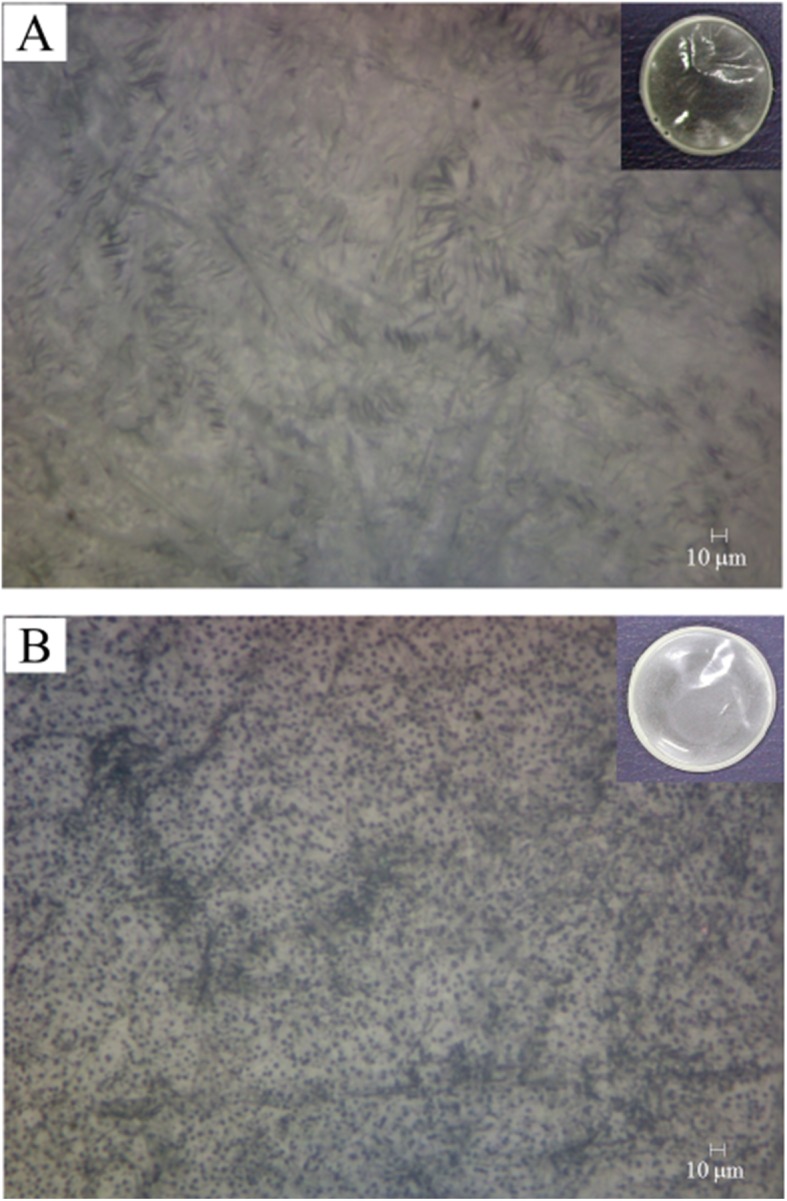
Video-microscopic images of the solvent-cast gemcitabine-incorporated polyurethane (GPU) films. The gross images without magnification are shown in the insets. The GPU film with 2% gemcitabine loading was transparent without any indication of drug aggregate (**a**). The film with 10% gemcitabine loading was translucent, and gemcitabine aggregate particles, with a size of a few micrometer, were seen on video microscopy (**b**)

The GPU film was completely transparent up to a drug load of 2% (w/v), but the transparency decreased with increase in the drug loading, and the GPU film with 10% (w/v) drug load was translucent (Fig. 2). Video-microscopic examination showed some irregular dark valley-like structures for the GPU film with 2% drug load as shown in Fig. 2a. These structures, however, also appeared in the background of the small black dots for the GPU film with 10% drug load (Fig. 2b). We also observed irregular structures for the PU film without drug loading and concluded that these structures are not drug-related, but that they are the results of light reflection from an uneven film surface (Additional file 1: Figure S2). Black dot structures with a size of a few micrometers were seen scattered randomly across the film when the drug load was increased to 10% (w/v) (Fig. 2b). Since these dots were not seen for GPU films with lower drug-load, we interpret these black dots as aggregates of gemcitabine molecules formed during the solvent evaporation process.

### Morphology of the silicone-GPU interface

**Fig. 3:**
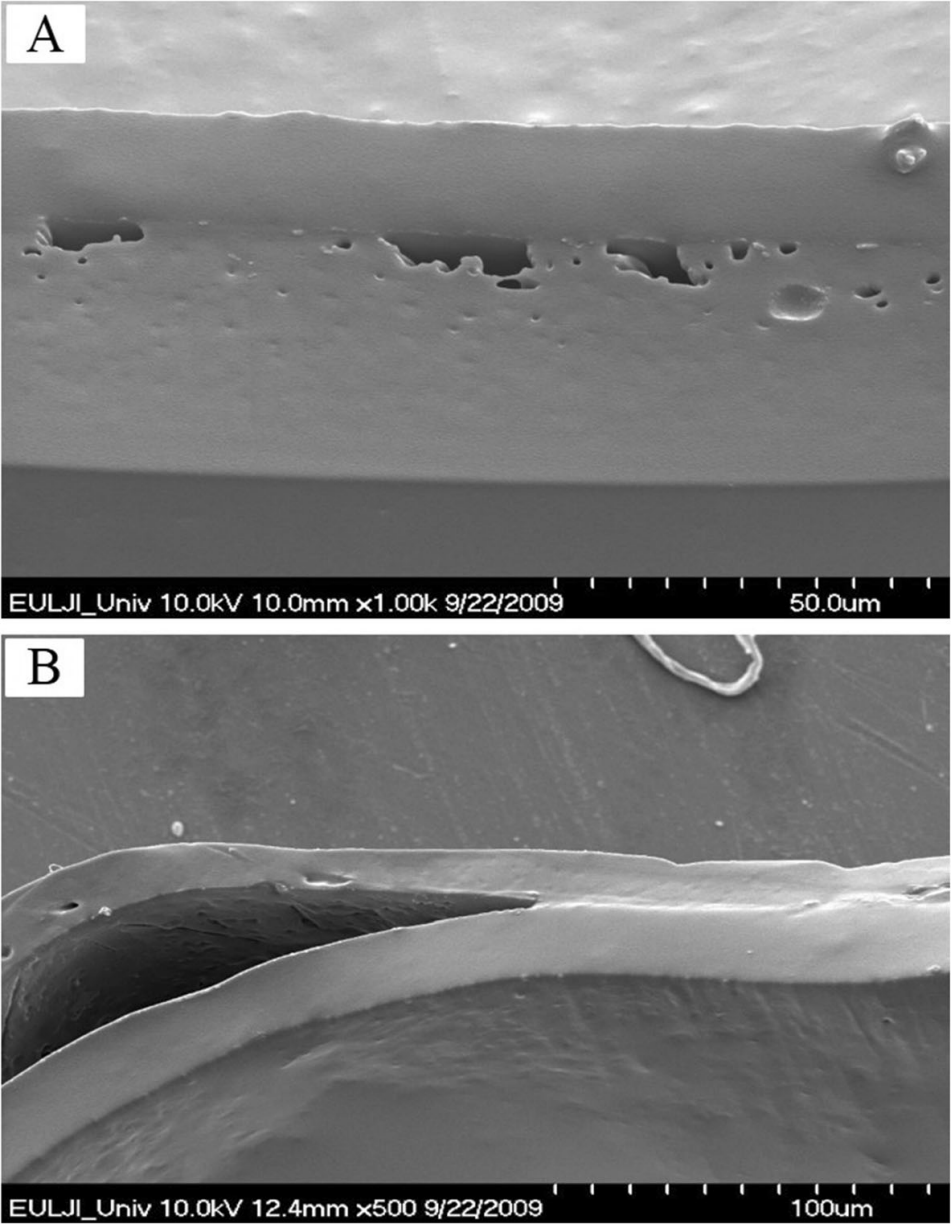
Scanning electron microscopic images of the Si-GPU-PU film interface before and after the drug release study. Unfilled voids are seen at the interface before drug release study (**a**), and separation between the two films increased upon immersion in a phosphate-buffered saline solution for 1 day in the drug-release study (**b**)

The scanning electron microscopic images of the interface between the silicone and the GPU in a Si-GPU-PU film are shown in Fig. 3. The interface between the PU and GPU layers was not discernible, but the film before drug-release study showed incomplete adhesion between the two Si-GPU polymer layers; there were unfilled voids, as large as 20 μm, between the two layers (Fig. 3a). The holes eventually led to a partial separation of the two layers after immersion in the PBS buffer for 1 day (Fig. 3b).

### In vitro drug release study

**Fig. 4:**
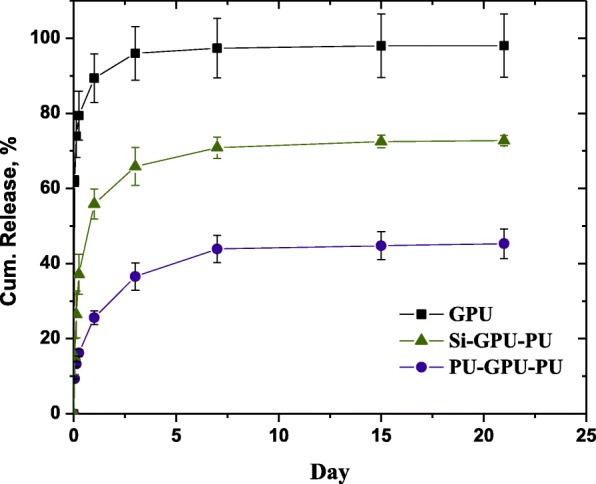
Cumulative release of gemcitabine from gemcitabine-incorporated polyurethane films. About 90% of the load was released in a day from the GPU film, but the cumulative release in a day decreased to 26% when the GPU film was enveloped by PU barrier films (PU-GPU-PU). When the GPU film was sandwiched in between the silicone and polyurethane barrier films, 56% of the drug load was released in a day (Si-GPU-PU)

The in vitro release behavior of gemcitabine from the gemcitabine-incorporated films is shown in Fig. 4. About 74% of the drug was released in 3 h in the case of the GPU film, and 90% of the drug load was released in a day. The drug release was virtually complete in 3 days. The drug release was much slower when the drug-loaded film was enveloped with a polyurethane film; only 26% of the drug load in the PU-GPU-PU film was released in a day and more than 50% of the drug load remained unreleased after 1 week. The drug release behavior of Si-GPU-PU was intermediate between those of the GPU and PU-GPU-PU films; 57% drug load was released in a day and 30% of the load remained unreleased after 1 week.

## Discussion

**Fig. 5:**
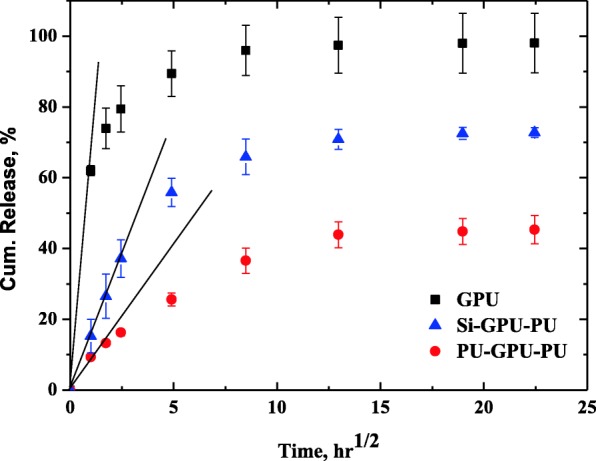
Cumulative release of gemcitabine from GPU films plotted against “square root of time.” The linear region of cumulative release relative to the “square root of time” decreased when the GPU film was sandwiched in between the silicone and polyurethane films (Si-GPU-PU) or two homo polymer polyurethane films (PU-GPU-PU)

The release behavior of a drug in a polymer matrix with drug concentration much larger than the drug solubility in the polymer matrix could be explained by a Higuchi model, where most of the drug is dispersed as a solid aggregate in the polymer matrix, with only a small portion dissolved in the matrix [13, 14]. The video microscopic image of 10% (w/v) GPU films in Fig. 2 shows that the GPU films with a gemcitabine load of 10% (w/v) clearly had dispersed drug aggregate particles. The Higuchi model assumes that the solid drug aggregate particles on the surface of the film dissolve first, and that the solid drug aggregate particles in the next layer are dissolved by water coming from outside the polymer matrix through the spaces or the micro-channels created by previous dissolution of the drug aggregate particles [14]. This model predicts a “square root of time” release kinetics. We plotted the cumulative release of gemcitabine from three polymer films against square root of time in Fig. 5. The cumulative release of gemcitabine from the GPU film was indeed linearly proportional to the “square root of time” initially, but the slope decreased with time. The decrease in the slope with the GPU film could be because of the isolated aggregate particles that were farther from the neighboring particles on average and the molecularly dissolved gemcitabine molecules in the polyurethane matrix that did not aggregate to particles. In both the cases, the drug release takes more time because the drug molecules have less chance of contact with the incoming water molecules. The Higuchi model is expected to be effective up to 60% drug release, and the slope of the PU-GPU-PU film in Fig. 5 indicates that the effective range is much smaller when the drug loaded film is covered by barrier films. This result indicates that the formation of pores and channels after dissolution of aggregate drug particles becomes difficult as the distance from the circumference of the drug-loaded film increases. Baskaran et al. studied doxycycline release behavior from a core-shell type nanofiber-covered trachea stent, and reported that the Higuchi model is well-suited when the initial drug concentration in the matrix is much higher than drug solubility in the matrix [15]. The presence of aggregate particles in GPU films, shown in Fig. 3b, evidences that the drug concentration in GPU films for in vitro release study is much higher than the gemcitabine solubility in PU film.

The dramatic decrease of the initial burst in the PU-GPU-PU film, compared to the one for the GPU film shown in Fig. 4, is an indication that the presence of a pure polymer film envelope around the drug-loaded film is an effective method for slowing down rapid drug release. The initial burst (the drug released in a day) was 90% for the GPU film but it decreased to 26% for the PU-GPU-PU film. Moreover, almost 40% of the drug load remained undissolved after 1 week. These results indicate that it is much more difficult for water molecules to get to the drug aggregate particles when the drug loaded GPU film is enveloped by barrier PU films both on the top and on the bottom. We postulate that 16% of the initial burst in the PU-GPU-PU film reflects the dissolution of the drug aggregate particles on or near the circumference of the circular drug-loaded GPU film. After the initial burst of the drug aggregate particles in the circumference of the GPU film, those inside the circular GPU film could be accessed only by the water molecules invading from the circumference, as water penetration from the top and bottom surfaces was blocked by the PU films. The distance of the center of the GPU film from the circumference was 7500 μm, whereas the distance from the top or bottom of a GPU film was less than 10 μm. Thus, we can expect much slower drug release from the GPU film when the top and bottom surfaces are blocked by the PU film. The fact that about 40% of the drug remained undissolved after 1 week is an indication that the polyurethane envelope at the top and bottom of the GPU film was effective in decreasing the initial burst of the drug release or for controlling the rapid release of the drug. The very slow drug release from the PU-GPU-PU film in the plateau region after 1 week might not be a problem in clinical situations, as polyurethane is known to be biodegradable [16, 17], and the gemcitabine release from the PU-GPU-PU film should be higher than that suggested in Fig. 4.

The drug release behavior of Si-GPU-PU film was in the middle, in between those of GPU film and PU-GPU-PU film. About 37% of the drug was released in a day and about one-third of the drug load remained in the film after 1 week. These results indicate that only half of the envelope was effective for controlling the rapid gemcitabine release, and that the silicone envelope was ineffective in controlling the gemcitabine release, as evidenced by the partial separation of silicone layer upon immersing the film in PBS buffer, as shown in Fig. 2b. Water can penetrate the partially separated cracks in the silicone-GPU interface, and the drug aggregate particles can easily be dissolved by water invading the GPU matrix from the silicone-PU interface.

We tested the silicone barrier in this study, because the polyurethane films covered onto a stent are frequently found perforated after implantation [16], whereas silicone film is known to be more biodurable compared to the polyurethane film [17]. Our intention was to block the release of anticancer drug molecules into the lumens of intestinal tract with the silicone barrier film. The results obtained in this study, however, indicate that we need a more robust method for attachment of the silicone film and the gemcitabine-loaded polyurethane film that is stable in water environment.

Maintaining strong adhesion through a physical attractive force between the two different polymers that are stable in aqueous environments is a difficult task, even after surface activation through plasma treatment. The hydrophilic functional groups can be integrated onto a polymer surface by plasma treatment, and oxygen containing functional groups (e.g. –C–O, −C=O) as well as nitrogen containing groups (e.g. NH_2_, N–C=O) can be introduced on the surface of a polymer using the ambient air under atmospheric pressure, with plasma treatment [18–20]. The introduction of hydrophilic functional groups is known to improve the wettability and adhesion properties of the polymer. As evidenced in Fig. 3a, the plasma treatment indeed improved the adhesion of polyurethane onto the silicone; the two layers were easily separated by hand without the plasma treatment, but they were inseparable when the polyurethane was solvent-casted after treatment of the silicone with plasma. The improved hydrophilicity of the silicone surface must have helped the adhesion of polyurethane chains onto the silicone surface, but the improvement in adhesive force between the two layers did not persist in the water environment, as shown in Fig. 3b, where micro-scale separation is evident after immersion in water for 1 day. We also observed partially-separated and fully-separated films after the 21-day drug release study. More studies are warranted to develop an attachment method between silicone and drug-loaded polyurethane film.

## Conclusions

Gemcitabine has been an effective anti-cancer drug for systemic administration, but local delivery of gemcitabine for improved efficacy in malignant tumor control has yet to be realized. We studied GPU films layered in between polyurethane homo polymer films and silicone and polyurethane films. The layering strategy with polyurethane homopolymer films was successful, but a stronger adhesive force was required to layer a silicone film over the GPU film to retard the gemcitabine release rate.

## Supplementary information

**Additional file 1: Figure S1.** High pressure liquid chromatogram of gemcitabine released from a GPU film. **Figure S2.** Video-microscopic image PU film without gemcitabine loading. Sideway illumination was used to show the unevenness of the surface.

## Data Availability

For data requests, please contact the authors.

## Abbreviations

GPU: Gemcitabine-incorporated polyurethane
PU: Polyurethane
