# Supplementary material for: Gemcitabine-incorporated polyurethane films for controlled release of an anticancer drug
Source: Biomater Res. 2019 Nov 20;23:19. doi: 10.1186/s40824-019-0169-7 (PMC6865006; doi:10.1186/s40824-019-0169-7)
Supplement: Supplementary file 1 — Additional file 1: Figure S1. High pressure liquid chromatogram of gemcitabine released from a GPU film. Figure S2. Video-microscopic image PU film without gemcitabine loading. Sideway illumination was used to show the unevenness of the surface. [file 40824_2019_169_MOESM1_ESM.docx]

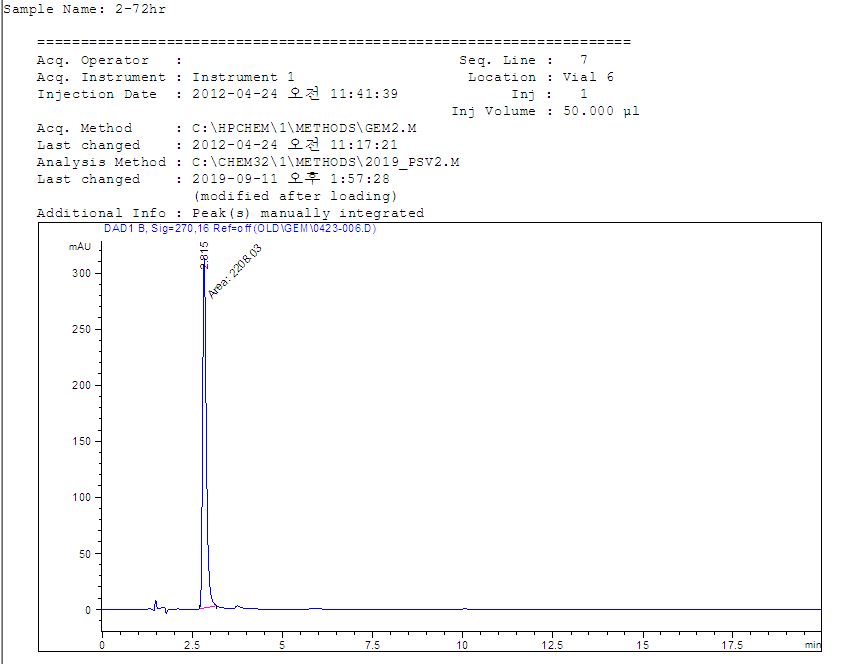


Fig S1. High pressure liquid chromatogram of gemcitabine released from a GPU film.


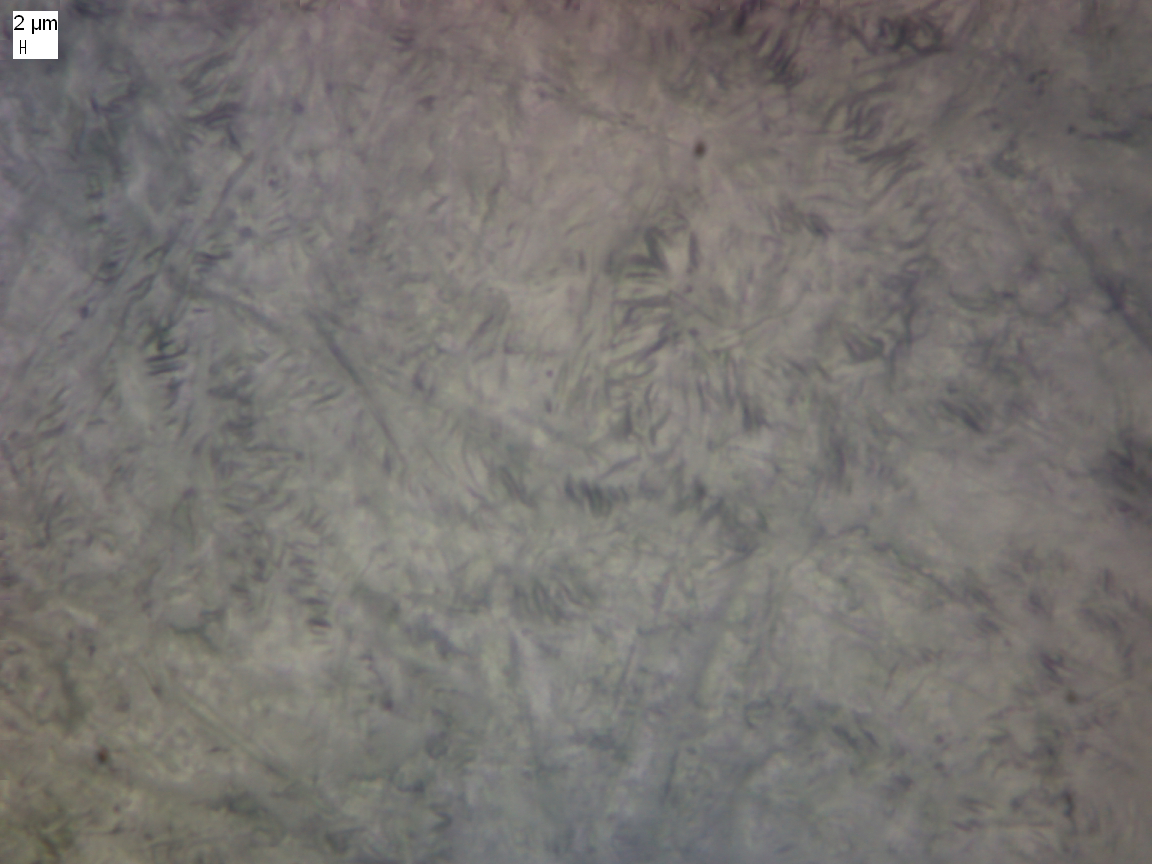


Fig S2. Video-microscopic image PU film without gemcitabine loading. Sideway illumination was used to show the unevenness of the surface.
